# Autoimmune Pulmonary Alveolar Proteinosis Triggered by Salazosulfapyridine in a Patient With Rheumatoid Arthritis: A Case Report

**DOI:** 10.7759/cureus.99760

**Published:** 2025-12-21

**Authors:** Tomoyuki Ikeuchi, Mitsuhiro Yamamoto, Hirokazu Touge, Naoto Burioka, Akira Yamasaki

**Affiliations:** 1 Department of Respiratory Medicine, National Hospital Organization (NHO) Yonago Medical Center, Yonago, JPN; 2 Division of Respiratory Medicine and Rheumatology, Department of Multidisciplinary Internal Medicine, School of Medicine, Faculty of Medicine, Tottori University, Yonago, JPN

**Keywords:** anti- granulocyte-macrophage colony-stimulating factor antibodies, autoimmune pulmonary alveolar proteinosis, rheumatoid arthritis, salazosulfapyridine, sargramostim

## Abstract

A 91-year-old woman diagnosed with rheumatoid arthritis (RA) in her 40s achieved disease stabilization after salazosulfapyridine (SASP) treatment. In May 2025, chest computed tomography (CT) revealed bilateral ground-glass opacities with interlobular septal thickening, presenting a crazy-paving pattern. She was diagnosed with autoimmune pulmonary alveolar proteinosis (aPAP) based on positive serum anti-granulocyte-macrophage colony-stimulating factor (GM-CSF) antibodies and periodic acid-Schiff (PAS)-positive fluid accumulation in the alveoli identified on transbronchial lung biopsy (TBLB). The development of aPAP is considered extremely rare in patients with RA, as the disease is typically characterized by elevated GM-CSF activity, creating a pathological paradox. Previous reports have revealed that almost all patients with aPAP and RA receive SASP treatment. We hypothesized that aPAP associated with RA developed due to a dual suppression of GM-CSF: drug-induced inhibition of GM-CSF at the alveolar level and acquired production of anti-GM-CSF antibodies.

## Introduction

Pulmonary alveolar proteinosis (PAP) is a disorder in which pulmonary surfactant abnormally accumulates in the alveolar spaces due to disruption in surfactant production or clearance processes, resulting in respiratory failure. Based on etiology, PAP is categorized as autoimmune, secondary, congenital, or unclassified. Autoimmune pulmonary alveolar proteinosis (aPAP) represents approximately 90% of all PAP cases [1]. In patients with aPAP, granulocyte-macrophage colony-stimulating factor (GM-CSF) in the lungs is neutralized by autoantibodies, resulting in impaired maturation of alveolar macrophages and subsequent accumulation of pulmonary surfactant. In contrast, in patients with rheumatoid arthritis (RA), GM-CSF is overexpressed as an inflammatory cytokine, and a monoclonal antibody against GM-CSF receptor is being investigated as novel therapeutic agents [2]. Thus, aPAP and RA represent contradictory pathological conditions: aPAP is characterized by insufficient GM-CSF activity, whereas RA is characterized by excessive GM-CSF. Therefore, the coexistence of both diseases is extremely rare [3]. This patient had comorbid RA and underwent salazosulfapyridine (SASP) treatment. Herein, we present a case of aPAP diagnosed in a patient with RA, discussing the diagnostic approach and exploring a potential link to SASP therapy. We reviewed previously reported cases of aPAP associated with RA and discussed the presumed pathogenic mechanisms.

## Case presentation

A 91-year-old woman presented with dyspnea upon exertion, with ground-glass opacities in both lung fields. She had been diagnosed with RA in her 40s. The patient’s condition stabilized with SASP treatment, and she was being managed at a local clinic. During a routine visit in May 2025, chest radiography and computed tomography (CT) revealed ground-glass opacities in both lung fields. A follow-up chest CT performed three weeks later showed enlargement of the opacities. Dyspnea on exertion persisted; however, the patient had no other accompanying symptoms, including cough, sputum production, or fever. the patient was referred to our department for further investigation. She had no history of smoking or exposure to dust.

On physical examination, the patient had a blood pressure of 120/71 mmHg, a heart rate of 73 bpm, a respiratory rate of 20/min, an oxygen saturation of 93% on room air, and a temperature of 36.8°C. Her breath sounds were clear, and her fingers and limb joints showed no tenderness or deformities.

**Figure 1:**
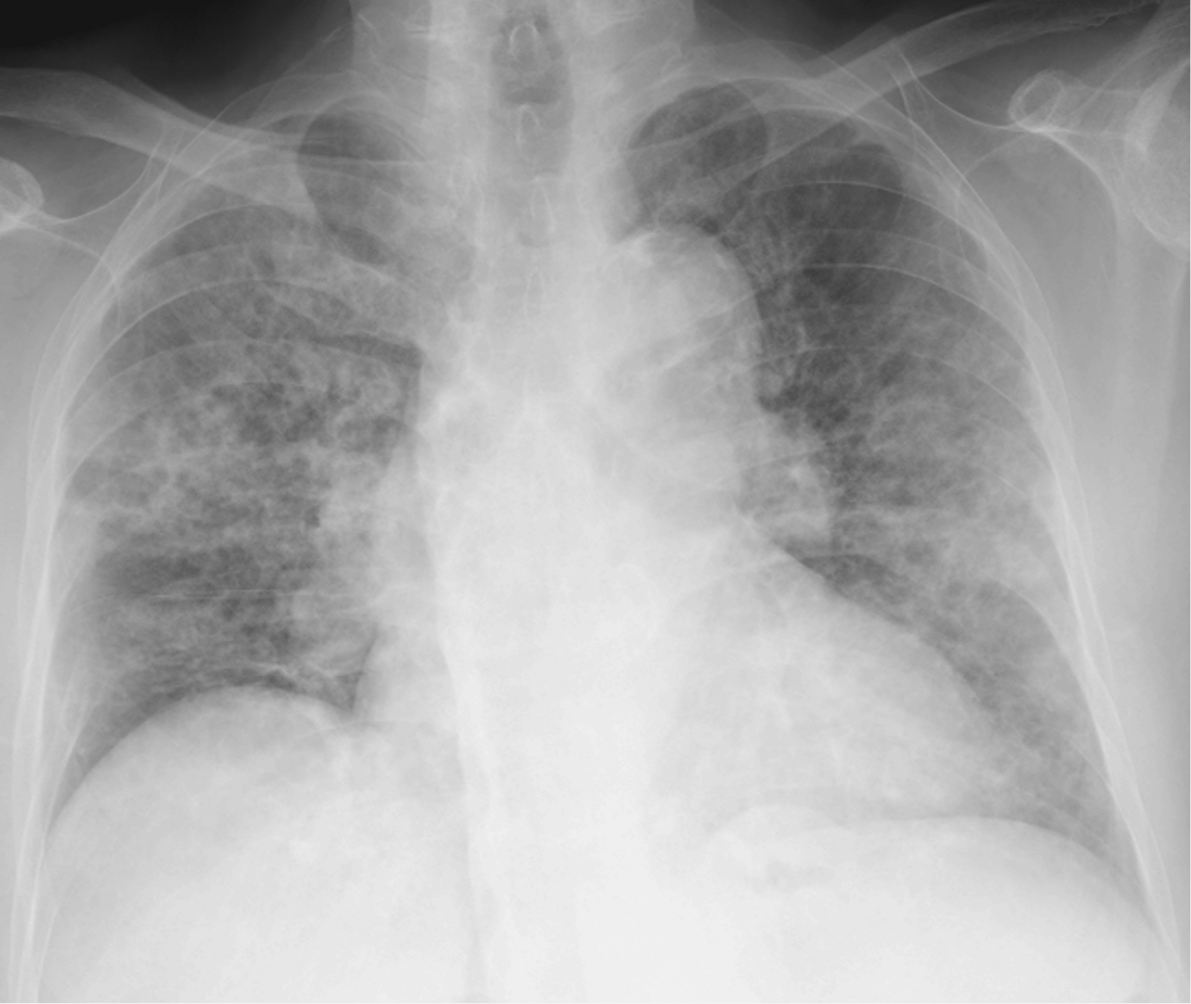
Chest X-ray at the initial visit Chest X-ray showed bilateral ground-glass opacities.

**Figure 2:**
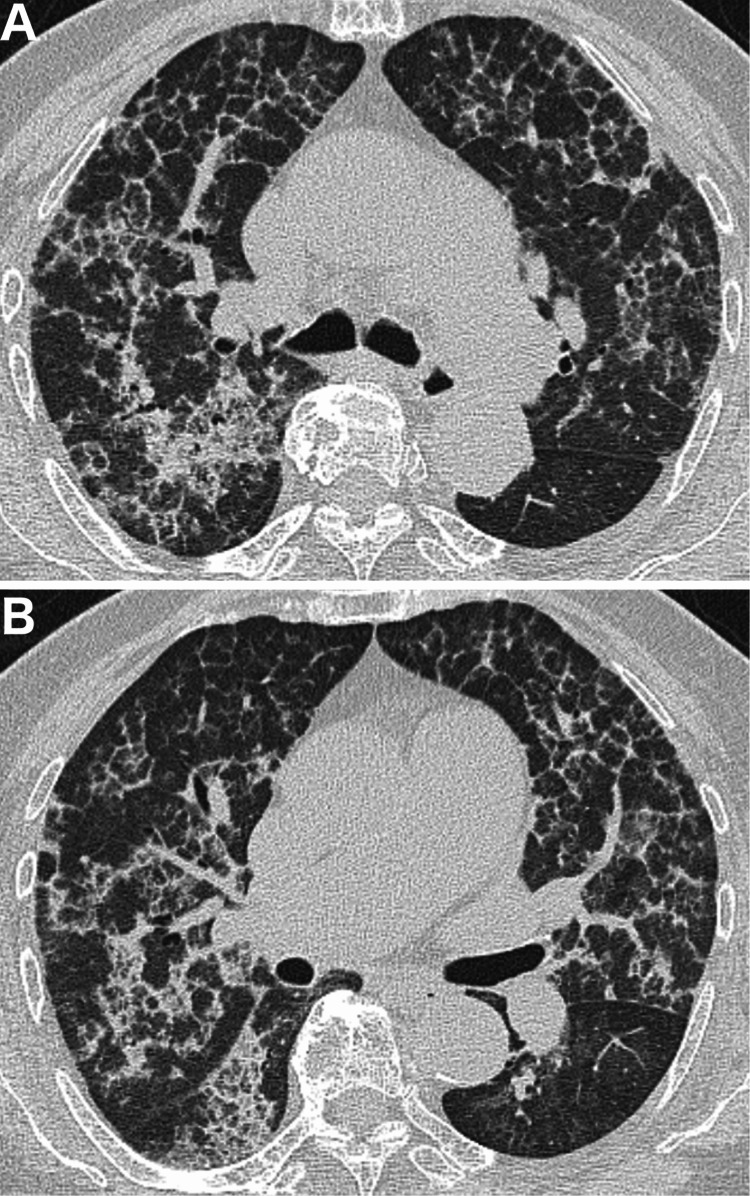
Chest CT scan at the time of diagnosis Chest computed tomography (CT) revealed interlobular septal thickening against a background of ground-glass opacities, presenting a crazy-paving pattern. A. CT slice at the carinal level. B. CT slice at the middle lobe and lingular level.

Chest radiography revealed diffuse reticular ground-glass opacities in both lungs (Figure 1). Chest CT revealed bilateral ground-glass opacities with interlobular septal thickening, presenting a crazy-paving pattern (Figure 2). The laboratory test results are presented in Table 1. Complete blood count, serum biochemistry, and C-reactive protein (CRP) levels were within normal limits. Sialylated carbohydrate antigen KL-6, pulmonary surfactant protein D (SP-D), and pulmonary surfactant protein A (SP-A) levels were elevated. Rheumatoid factor (RF) levels were elevated, whereas matrix metalloproteinase-3 (MMP-3) levels were within the normal range. RA disease activity was well controlled, with a DAS28-CRP level of 1.2. Anti-GM-CSF antibodies were detected using a simple serum diagnostic kit. Arterial blood gas analysis revealed mild respiratory failure and elevated A-aDO₂ levels. Pulmonary function testing revealed a decrease in %DLCO (diffusing capacity of the lungs for carbon monoxide).

**Table 1:** Laboratory data

Laboratory test (blood)	Unit	Result	Normal Range
Complete blood count			
White blood cells	1000/μL	4.6	3.3-8.6
Red blood cells	million/μL	3.58	3.86-4.92
Hemoglobin	g/dL	11.0	11.6-14.8
Platelets	'000/μL	174	158-348
Neutrophils	%	50.0%	36.0-69.5
Lymphocytes	%	18.5%	20.8-52.7
Monocytes	%	4.2%	4.5-10.6
Eosinophils	%	0.3%	0.5-8.8
Basophils	%	0.2%	0.2-1.7
Biochemistry			
Sodium	mmol/L	144	138-145
Potassium	mmol/L	3.9	3.6-4.8
Chloride	mmol/L	110	98-107
Creatinine	mg/dL	0.6	0.46-0.79
Blood urea nitrogen (BUN)	mg/dL	14	8-20
Glucose	mg/dL	118	73-109
Protein, total	g/dL	7.7	6.6-8.1
Albumin	g/dL	3.8	4.1-5.1
Globulin	g/dL	3.4	2.2-3.4
Albumin/globulin Ratio		1.1	1.32-2.23
Bilirubin, total	mg/dL	0.5	0.4-1.5
Alanine transaminase	U/L	13	7-23
Aspartate aminotransferase	U/L	23	13-30
Alkaline phosphatase	U/L	107	38-113
Serum			
CRP	mg/dL	0.02	0-0.14
Sialylated carbohydrate antigen KL-6	U/mL	1200	0-500
Pulmonary surfactant protein D (SP-D)	ng/mL	276	<110
Pulmonary surfactant protein A (SP-A)	ng/mL	64.7	0-43.8
Anti-granulocyte-macrophage colony-stimulating factor (GM-CSF) antibody		positive	negative(<3.5U/mL)
β-D-glucan	pg/mL	25.6	0-20
IgG	mg/dL	1349	861-1747
Rheumatoid factor (RF)	IU/mL	222	0-15
Matrix metalloproteinase-3 (MMP-3)	mg/mL	55.0	17.3-59.7
Arterial blood gas analysis (room air)			
pH		7.41	7.35-7.45
PaCO_2 _(partial pressure of carbon dioxide)	Torr	37.4	35-45
PaO_2_ (partial pressure of oxygen)	Torr	61.9	75-100
HCO_3_^-^	mmol/L	23.1	22-26
A-aDO_2_	Torr	41.1	≤10
Pulmonary function test			
FVC (Forced vital capacity)	L	1.45(85.9%)	1.48(reference value)
FEV1 (Forced expiratory volume in one second)	L	1.09(103.8%)	1.05(reference value)
FEV1% (FEV1/FVC)	%	75.2	≥70
%DL_CO_ (carbon monoxide diffusing capacity)	%	77.4	80-120

**Figure 3:**
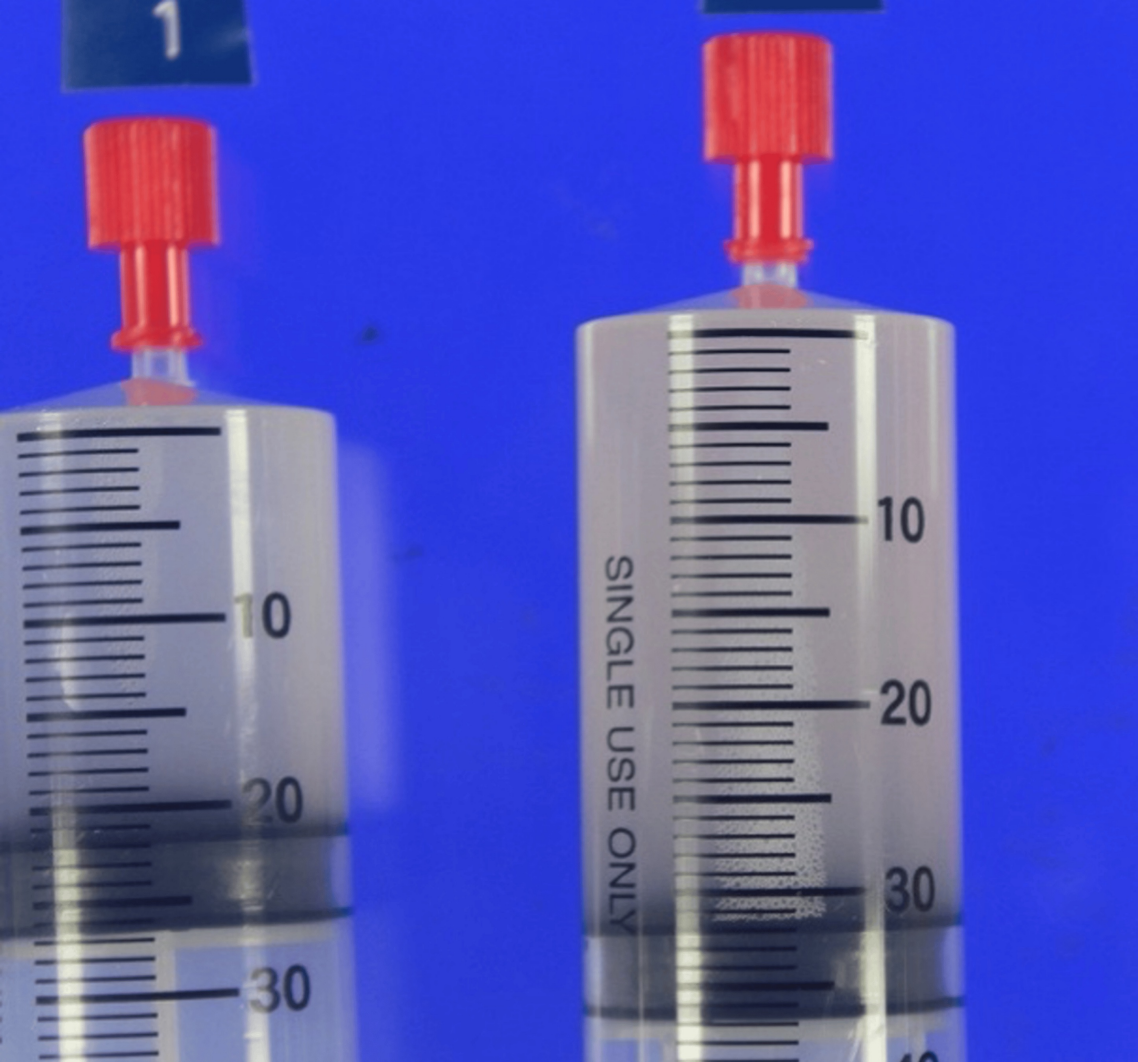
Appearance of the BAL fluid The bronchoalveolar lavage fluid (BALF) was milky white. Cytological examination revealed benign findings, with scattered ciliated columnar epithelial cells and alveolar macrophages against a background of periodic acid-Schiff (PAS)-positive material.

**Figure 4:**
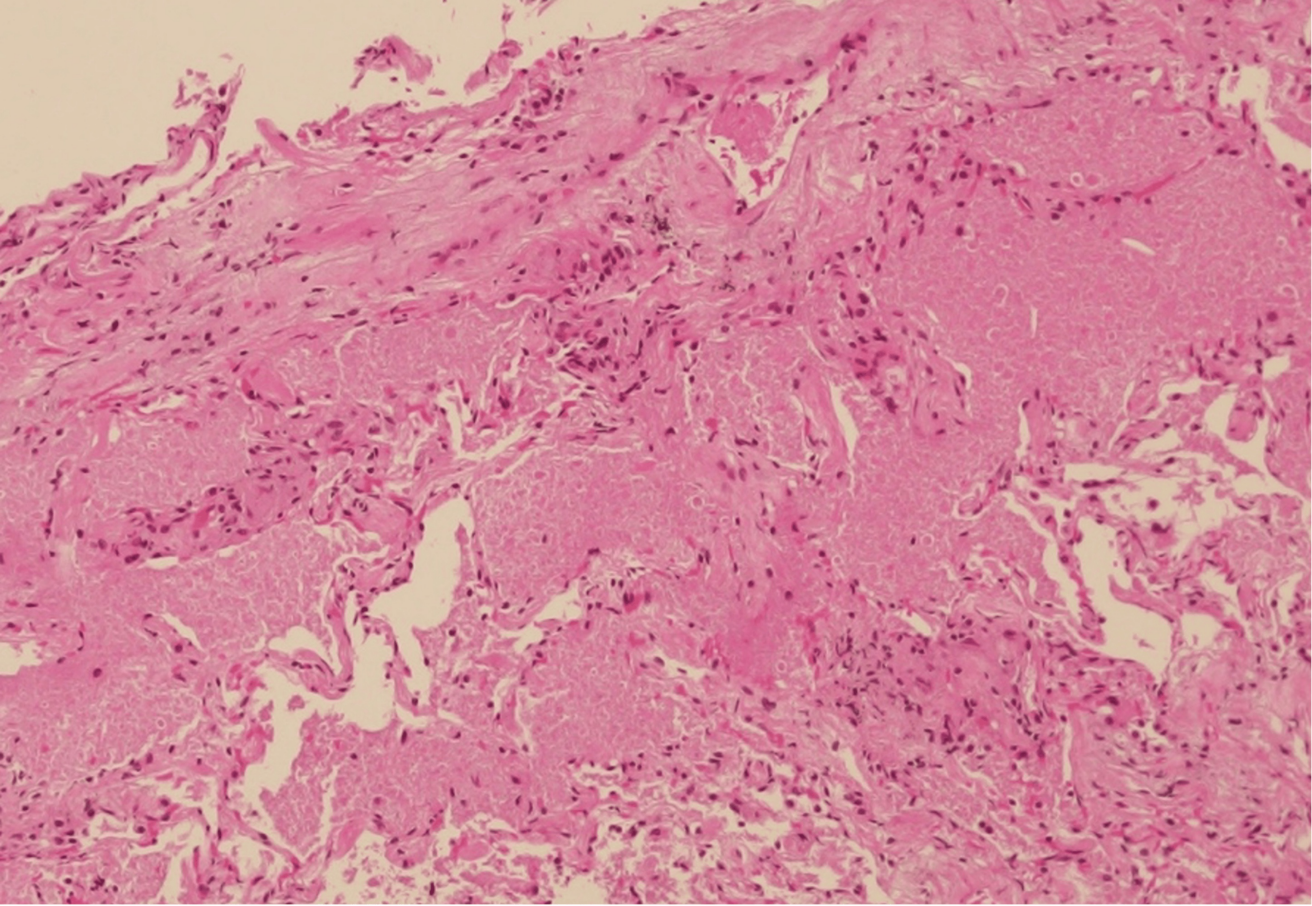
Histopathology of the lung (lt. B3, hematoxylin and eosin (H&E) stain) The alveolar structure remains intact. Pale eosinophilic periodic acid-Schiff (PAS)-positive material was observed in the alveolar spaces.

Bronchoalveolar lavage (BAL) was performed in the left B^4b^ and transbronchial lung biopsy (TBLB) was obtained from the left B^3^. The BAL fluid appeared turbid and milky white (Figure 3). The recovery rate was 98/150 mL (65%). Total cell count was 277 cells/μL; a cell differential could not be performed due to significant cell disruption, a finding often associated with PAP due to fragile, lipid-laden macrophages. The mean CD4/CD8 ratio was 0.69. Cultures for common bacteria and acid-fast bacilli were negative. TBLB (Figure 4) showed preserved alveolar architecture, with pale eosinophilic material positive for periodic acid-Schiff (PAS) staining within the alveolar spaces.

The patient was diagnosed with aPAP based on the presence of anti-GM-CSF antibodies. Inhalation therapy with sargramostim, a GM-CSF agent, was initiated in July 2025. Since RA was well controlled with SASP treatment, SASP therapy was continued. Chest CT scan was performed approximately one month later, in August, showed improvement in ground-glass opacities in the right lower lobe. Arterial blood gas analysis on September revealed PaO₂ of 64.2 Torr and A-aDO₂, of 38.8 Torr, indicating slight improvement. The planned treatment regimen, one week of inhalation therapy followed by one week off, will be repeated for up to 12 cycles [4].

## Discussion

aPAP and RA represent contradictory pathological conditions, and their coexistence is extremely rare. A PubMed search for cases of concurrent aPAP and RA identified only four previously reported cases (Table 2) [3,5,6]. Including the present case, we were able to identify five cases. Of these five cases, four patients received SASP for RA treatment. In one case treated with leflunomide and prednisone [5], the anti-GM-CSF antibody test was negative, and drug-induced PAP was suspected.

**Table 2:** Reported cases of aPAP with RA aPAP: Autoimmune pulmonary alveolar proteinosis; RA: rheumatoid arthritis; LEF, leflunomide; PSL, prednisone; SASP, salazosulfapyridine; MTX, methotrexate; SOB, shortness of breath; DOE, dyspnea on exertion; DC, dry cough; WLL, whole-lung lavage; GI, GM-CSF inhalation; CS, systemic corticosteroid

Reporting year	Reference No.	Sex	Age	RF (IU/mL)	Duration of RA treatment	DMARDs	Symptoms	anti-GM-CSF antibody (U/mL)	Treatments
2006	Wardwell et al., 2006 [5]	Male	42	Not listed	One month	LEF+PSL	SOB	Negative	WLL, withdrawal of LEF
2017	Ito et al., 2017 [6]	Female	65	29.3	Five years	SASP+MTX	DOE, DC	26.1	WLL, GI, CS
2017	Ito et al., 2017 [6]	Female	68	238.3	26 years	SASP	DOE	42.3	WLL, GI
2020	Hashimoto et al., 2020 [3]	Male	70	110.5	Five years	SASP	DC	40.5	Expectorant
-	Present case	Female	91	222.0	45 years	SASP	DOE	Positive (>3.5)	GI

Orally administered SASP is metabolized by intestinal bacteria into sulfapyridine (SP) and 5-aminosalicylic acid. The therapeutic effect in RA is primarily attributed to SP, which is readily absorbed, and previous clinical trials [7] have demonstrated that SP alone achieves efficacy comparable to that of SASP. SASP has multiple effects on the immune system, which are thought to include the suppression of antibody production through B-cell inhibition, suppression of inflammatory cytokines, and modulation of signal transduction pathways, including inhibition of nuclear transcription factors such as NF-κB (nuclear factor kappa-light-chain-enhancer of activated B cells) [8-10]. We hypothesize that SASP, through its known inhibition of NF-κB and other immunomodulatory pathways, may indirectly suppress GM-CSF signaling or production within the alveolar microenvironment. Clinical data observations indirectly supporting this association include several reported cases of SASP-induced agranulocytosis that recovered rapidly with GM-CSF administration [11].

Previous studies have measured SP and SASP in the synovial fluid of patients with RA treated with SASP [12,13]. SP and SASP are systemically well absorbed from the colon and are presumed to have a high tissue distribution. The immunomodulatory effects of SP and SASP on the lungs may suppress GM-CSF activity within the alveoli. We propose a 'dual-hit' pathogenesis: first, SASP may partially suppress local GM-CSF activity, creating a permissive environment; second, the acquired production of anti-GM-CSF antibodies provides the final, critical hit that neutralizes GM-CSF sufficiently to cause impaired surfactant clearance and clinical aPAP. We speculate that under these circumstances, anti-GM-CSF antibodies were acquired, leading to dual suppression of GM-CSF, and a subsequent decline in its concentrations to a level that promoted pulmonary surfactant accumulation. Similar to other rare and fatal treatment-induced complications in chronic systemic disease, our case of SASP-triggered aPAP underscores that even standard, long-used therapies can uncommonly precipitate life-threatening conditions [14]. This reality demands heightened clinical vigilance to ensure timely diagnosis and intervention.

## Conclusions

As GM-CSF levels are elevated in patients with RA, the development of aPAP is rare. We reviewed previous reports, including the present case, on the development of aPAP in a patient with RA. Notably, four out of the five reported cases, including ours, were associated with SASP treatment, suggesting a potential drug-specific trigger rather than a mere association with RA itself. Because SASP exhibits GM-CSF-suppressive effects and high tissue distribution in the lungs, its administration suppresses GM-CSF activity within the alveoli. In addition, acquired anti-GM-CSF antibodies result in the dual suppression of GM-CSF, leading to the development of aPAP. When respiratory symptoms or pulmonary abnormalities develop in patients with RA receiving SASP, pulmonary alveolar proteinosis should be considered in the differential diagnosis. The present case was managed with inhaled sargramostim (GM-CSF agent) therapy. Further investigation is required to determine the most appropriate treatment for these pathological conditions, including SASP withdrawal.
